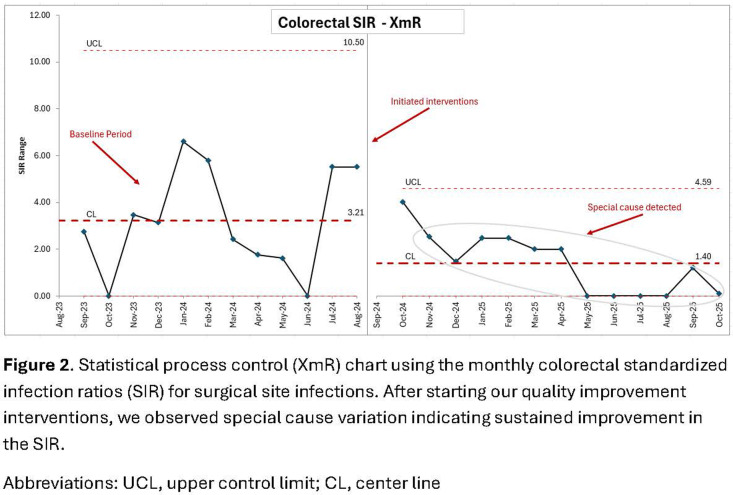# 58 Do Laws Regulating Legionella Growth Reduce Legionella Mortality? Evidence from Six Countries

**DOI:** 10.1017/ash.2026.10714

**Published:** 2026-06-23

**Authors:** Karen Techau, Graham Godwin, Nicole Carignan, Marissa Morales, Daisy Schwarz, Siyeh Gretzinger, Candace Skelton, Catherine Glaser, Avina Goel, April Kranz, Charles Finley, Jesse Jacob, Ari Reichstein, Jessica Howard-Anderson

**Affiliations:** 1 Emory University Hospital Midtown; 2 Emory Healthcare; 3 Emory University; 4 Emory University School of Medicine

## Abstract

**Background:** Surgical site infections (SSIs) following colorectal surgery are associated with increased morbidity, mortality, length of stay, and costs. During our hospital’s fiscal year (FY) 2024 reporting period (8/2023–7/2024), we had15 colorectal SSIs with a standardized infection ratio (SIR) of 2.43, indicating a need for focused improvement. **Methods:** In August 2024, we convened a multidisciplinary team in our 600-bed academic hospital, including perioperative nursing and education, infection prevention, anesthesia, colorectal surgery, infectious diseases, hospital epidemiology, quality and patient safety. After a gap analysis, the team implemented evidence-based interventions (Figure 1) guided by the Lean Six Sigma framework—Define, Measure, Analyze, Improve, and Control. The interventions included: 1) initiating apparent cause analysis (ACA) reviews for all colorectal SSIs with a multidisciplinary team; 2) standardizing colorectal surgery practices with wound protectors and a protocolized closing bundle (i.e. glove/gown change and closing tray); 3) creation of a perioperative antibiotic prophylaxis quick reference guide; and 4) clinical documentation improvement strategies for infections present at time of surgery (PATOS). We compared process measures (adherence to closing bundle, wound protector, and perioperative antibiotics) and outcome measures (number of SSI and SIR) between FY2024, FY2025 (8/2024-7/2025) and FY2026 (8/2025-10/2025). A run chart and Power BI were used for data visualization and metrics were reviewed monthly with the working group. A statistical process control (SPC) XmR chart was constructed retrospectively to assess process stability and detect special cause variation after the implementation of initiatives. **Results:** Compared to FY2024, we had fewer colorectal SSIs (15 vs 5) and a lower SIR (2.43 (95% confidence interval [CI] 1.41–3.91) vs 0.82 (95% CI 0.30-1.83) in FY2025 (Table 1). In the first quarter FY2026 we had two colorectal SSIs. Compared to FY2024, we observed increased adherence with the closing bundle (21%), wound protector use (13%), and perioperative antibiotics (4%) with continued increases in FY2026 (Table 1). Over time, early special cause variation was followed by stabilization with a centerline shift in SIR from 3.21 to 1.40 and upper control limit (UCL) reduction from 10.50 to 4.59 following implementation of quality improvement interventions (Figure 2). **Conclusion:** Iterative and continual process improvement with a multidisciplinary, engaged team significantly reduced colorectal SSIs at our hospital and improved compliance with key infection prevention measures. Ongoing efforts are focused on continued implementation of our key initiatives and sustainment.

**Figure:**
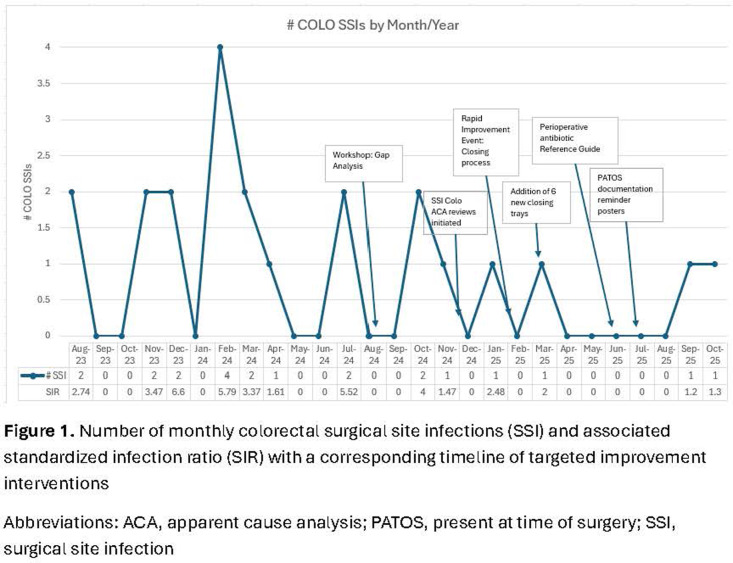

**Figure:**
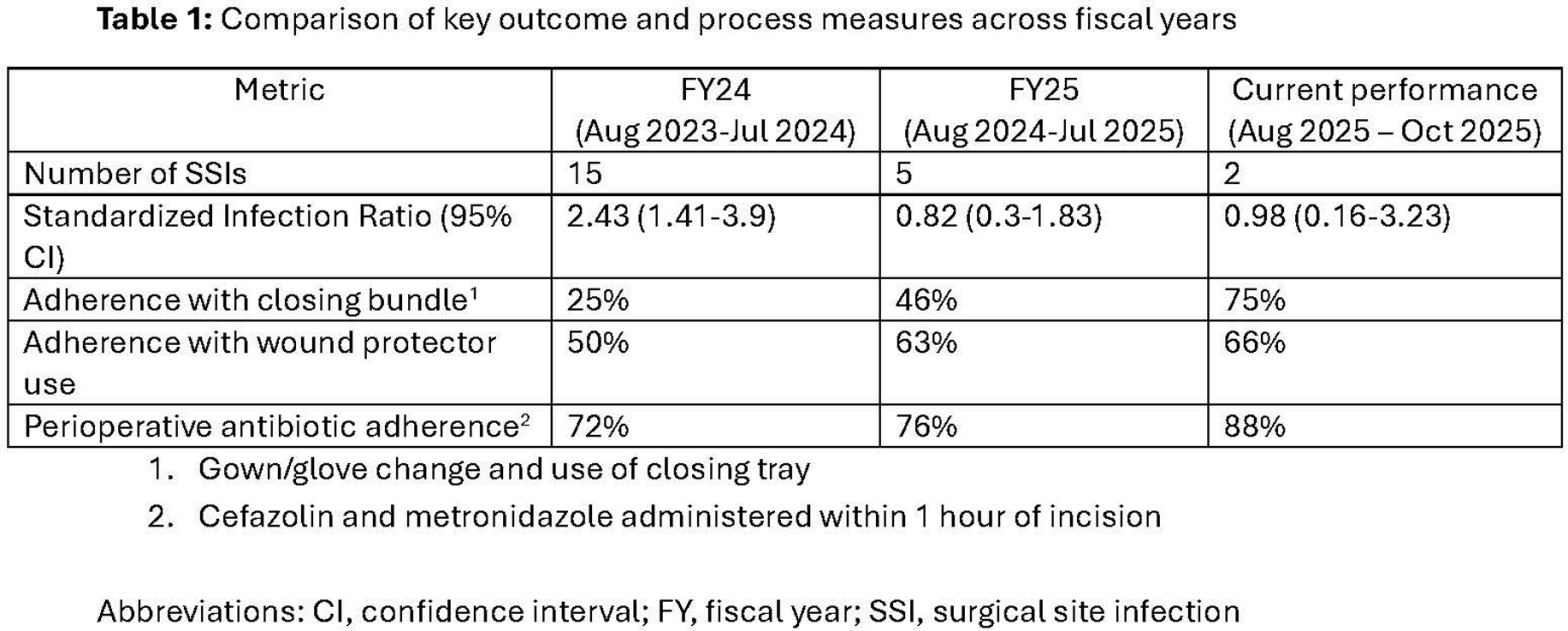

**Figure:**